# Fertilizer response and nitrogen use efficiency in African smallholder maize farms

**DOI:** 10.1007/s10705-018-9958-y

**Published:** 2018-11-15

**Authors:** Stephen M. Ichami, Keith D. Shepherd, Andrew M. Sila, Jetse J. Stoorvogel, Ellis Hoffland

**Affiliations:** 1 Department of Land Resource Management and Agricultural Technology, College of Agriculture and Veterinary Sciences, Faculty of Agriculture, University of Nairobi, P.O. Box 29053 - 00625, Kabete, Nairobi, Kenya; 2 World Agroforestry Centre (ICRAF), United Nations Avenue, Gigiri, P.O. Box 30677 - 00100, Nairobi, Kenya; 3 International Centre for Tropical Agriculture (CIAT-Kenya), DuduVille Lane off Kasarani Road, P.O. Box 823 - 00621, Nairobi, Kenya; 4 Soil Biology Group, Wageningen University, P.O. Box 47, 6700 AA Wageningen, The Netherlands; 5 Soil Geography and Landscape Group, Wageningen University, P.O. Box 47, 6700 AA Wageningen, The Netherlands

**Keywords:** Kenya, Meta-analysis, Nitrogen, Soil responsiveness, Spatial variability

## Abstract

Improving fertilizer recommendations for farmers is essential to increase food security in smallholder landscapes. Currently, blanket recommendations are provided across agro-ecological zones, although fertilizer response and nutrient use efficiency by maize crop are spatially variable. We aimed to identify factors that could help to refine fertilizer recommendation by analyzing the variability in fertilizer response (FR) and the agronomic nitrogen use efficiency (N-AE). A literature search for on-farm studies across Kenya and Sub-Sahara Africa (SSA), excluding Kenya, yielded 71 publications. The variability in FR was studied using a meta-analysis whereas key factors that influence FR and N-AE were studied with linear regression models. On average, the FR was 2, but it varied considerably from 1 to 28.5 (excluding outliers). In SSA, 18% of the plots were non-responsive plots with an FR < 1. The main factors affecting N-AE for Kenya were P-Olsen, silt content, soil pH, clay and rainfall, whereas only soil pH, exchangeable K and texture were important for SSA. However, our study indicates that available data on soil, climate and management factors could explain only a small part (< 33%) of the variation in FR and N-AE. Soil pH, P-Olsen, silt content, and rainfall had significant but low levels of power in explaining variation in FR and N-AE. Our findings indicate that strategies to refine fertilizer recommendation should include information on soil types and soil properties.

**Electronic supplementary material:**

The online version of this article (10.1007/s10705-018-9958-y) contains supplementary material, which is available to authorized users.

## Introduction

The population of Sub-Saharan Africa (SSA) is projected to increase up to 1.2 billion by 2050 (United Nations Population Division 2009; Ray et al. 2013). The increasing food demand of this growing population requires agricultural intensification with efficient fertilizer use. Current fertilizer recommendations in SSA are often only specified to the level of a region, for instance, an agro-ecological zone (AEZ) or administrative district (e.g., Mowo and Mlingano 1993; FURP 1994; Schnier et al. 1996). The fertilizer recommendations for these larger regions are commonly referred to as blanket fertilizer recommendations. However, environmental and management factors vary at short distances in the smallholder landscapes of SSA (Tittonell et al. 2005; Vanlauwe et al. 2011; Zingore et al. 2007; Stoorvogel and Smaling 1998; Tittonell et al. 2008). As a result, the blanket fertilizer recommendations are often considered to be of limited relevance to farmers (Tittonell et al. 2013).

The blanket fertilizer recommendations can only be refined if the factors that influence the variation in fertilizer response are known. This is increasingly urgent since an increasing number of farmers report a decreasing fertilizer response for staple food such as maize. Giller et al. (2006) introduced the concept of non-responsive soils: soils on which crops do not respond to mineral fertilizer application. However, the causes behind non-responsive soils are poorly understood. With a better understanding of factors that affect the variability in response to fertilizers, fertilizer recommendations can be improved. There is, however, a lack of studies that systematically identify key factors that affect the fertilizer response across smallholder farming systems of SSA. A recent meta-analysis pointed to the importance of secondary and micronutrient deficiencies in SSA in low fertilizer responses (Kihara et al. 2017). Multiple studies have shown that the fertilizer response varies across smallholder landscapes due to environmental (soil-related and climatic) and management factors. Zingore et al. (2007) demonstrate that the low level of soil organic carbon in maize fields of Zimbabwe led to a poor fertilizer response. Sileshi et al. (2008) attributed a high variability in rainfall amounts to low fertilizer response. Vanlauwe et al. (2016) observed that the poor fertilizer response in maize is a result of unbalanced soil fertilization. However, it remains unclear which are the key determinants (factors) of variability in fertilizer response.

Fertilizer recommendations (both type and amount of fertilizer) can be evaluated using indicators such as fertilizer response (FR) and agronomic nutrient use efficiency. The FR is defined as the incremental crop yield due to fertilization, independent of the quantity or the type of fertilizers applied. The FR is calculated as the ratio of fertilized crop yield and an unfertilized crop yield of a control plot. The FR is a useful concept for identifying, for example, “non-responsive soils”, i.e., soils in which no effect of fertilization is observed (Tittonell et al. 2007; Zingore et al. 2007; Njoroge et al. 2017). FR can also be used to evaluate the overall effect of fertilizer use across farms in a region. The agronomic nutrient use efficiency is a measure of the crop yield increase for a given amount of nutrient added (Dobermann et al. 2002) and can be used to evaluate the efficiency of a specific nutrient applied. For example, the agronomic nitrogen use efficiency (N-AE) is defined as the incremental crop yield per applied nitrogen (expressed in kg/kg).

Soil property maps of relevant variables to fertilizer management are increasingly becoming available (Hengl et al. 2015, 2017). These maps may help to get better insight in the spatial variability of nutrient concentrations (Antwi et al. 2016). However, these maps are generally at coarse spatial resolution and are only suitable for guiding recommendations at the regional scale but not at the farm level. Even though high throughput and cost-effective methods for soil analysis are also available (Shepherd and Walsh 2007; Shepherd 2010) most smallholder farmers do not have access to soil analyses services at the plot level. Knowledge of key factors that influence FR and N-AE is therefore critical for strategies aimed at improving nutrient management.

This paper aims to identify key factors that influence FR and N-AE to refine fertilizer recommendations for smallholder farmers. The specific objectives of this study were to: (1) quantify the variation in FR and N-AE, and (2) identify key environmental and management factors that influence variability in FR and N-AE. We employed a meta-analysis approach (Hedges et al. 1999; Borenstein et al. 2011) to analyze FR, and a regression analysis to understand the driving factors for FR and N-AE. This study focused on Nitrogen fertilization in maize across SSA with specific attention on Kenya. Maize is an important staple crop in the region and Nitrogen is found to be the most limiting nutrient in SSA (Nziguheba et al. 2009; Ma et al. 2016). A more detailed analysis was done for Kenya, because of the numerous agronomic studies conducted over a wide variation of environmental conditions.

## Materials and methods

### Literature search

A literature search on agronomic studies was conducted using Google Scholar, Mendeley, and Web of Science databases (1980–2016). All combination of the following terms were searched: ‘fertilizer application’, ‘maize yield’, ‘inorganic or mineral fertilizer’, ‘fertilizer response’, ‘smallholder’, ‘sub-Sahara Africa’, and ‘fertilizer treatment’ (e.g., ‘fertilizer response + maize yield + smallholder farm + sub-Sahara Africa’ or ‘fertilizer treatment + maize yield + smallholder farm’ or ‘fertilizer application + maize yield + smallholder farm’). Additional studies were searched in the reference lists of relevant publications. Publications consisted of peer-reviewed scientific articles, books, conference proceedings, and reports. Criteria used for obtaining a set of comparable studies for the analysis were: (1) maize was cultivated in monoculture, (2) the experiment was conducted on a smallholder farm in SSA, and (3) fertilizer treatments were randomly allocated to the plots. We selected treatments in which only inorganic N or combinations with inorganic P and or K were applied. Treatments in which additional organic fertilizers were applied were excluded. A systematic process for the selection of suitable fertilizer studies is presented in a flowchart in the Supplementary Material 2 (Fig. S1).

### Data extraction and treatment

Data on fertilizer treatments, crop yields, soils, climatic (agro-ecological zones) and management factors were extracted from the selected publications. A database was established with each record representing a treatment plot (Table 1).

**Table 1:** Variables and factors used in the analysis of the on-farm fertilizer experiments. (n = number of observations (paired treatments of control and fertilizer application), for SSA excluding Kenya)

Variables	Description	Units	n	
			Kenya	SSA
*Yield*				
Control	Mean maize yield for control plot	kg ha^−1^	202	256
Treatment	Mean maize yield for fertilized plot	kg ha^−1^	202	256
*Environmental factors (continuous)*				
Soil properties analysed prior to fertilizer trial	Soil pH,	–	194 (4)	230 (28)
	Total carbon (C)	g kg^−1^	192 (5)	181 (38)
	Total nitrogen (N)	g kg^−1^	155	216
	P-Olsen	mg kg^−1^	105 (19)	122 (22)
	P-Bray 2	mg kg^−1^	112	132
	P-Bray 1	mg kg^−1^	23	41
	Exchangeable K (Exch. K)	cmol kg^−1^	104 (34)	102 (48)
	Exch. Ca	cmol kg^−1^	78 (37)	71 (93)
	Exch. Mg	cmol kg^−1^	67	198
	Clay	%	109 (36)	152 (77)
	Sand	%	86 (38)	143 (89)
	Silt	%	96 (41)	161 (67)
Rainfall	Average per growing season	mm	198	220
Altitude	Height above sea level	m	186	85
*Environmental factors (categorical)*				
Soil orders (World Resource Base Reference Soil Groups)	Cambisols		22	–
	Nitisols		68	–
	Vertisols		6	–
	Ferralsols		81	24
	Luvisols		4	–
	Lixisols		41	
	Arcrisols		37	22
	Alfisols		12	4
	Phaeozems		–	8
	Alisols		8	–
Soil textural classes (USDA)	Clay		28	14
	Clay loam		21	–
	Loamy sand		2	8
	Sand		–	64
	Sandy clay		4	6
	Sandy clay loam		6	10
	Sandy loamy		31	55
Agro-ecological zone for Africa (Dudal 1980) and Kenya (Jätzold and Kutsch 2000)	Sub-humid (SSA)		18	–
	Humid (SSA)		6	–
	Lowlands		–	4
	Lower midlands		–	6
	Upper midlands		–	38
	Lower highlands (sub-humid)		–	6
	Lower highlands humid		–	6
*Management factors (continuous)*				
Fertilization rate	Amount of N applied	kg N ha^−1^	254	
*Management factors (categorical)*				
Nutrient applied	N only		149	235
	NPK		49	14
Manager	Farmer		159	234
	Researcher		81	63

n, number of observations; N, nitrogen; P, phosphorus; K, Potassium, the numbers in brackets are the number of observations that data imputation was performed for soil properties

Typically, experiments included various fertilizer treatments and or multiple seasons and sites. The data obtained from the studies contained multiple fertilizer treatment from a single experiment. Formally, these observations cannot be considered independent. However, López-López et al. (2014) showed that multiple entries from a single experiment are valid and can help to increase the precision of the analysis when using literature data. Controls were considered as treatments with no application of fertilizers (organic or inorganic). The data variables were harmonized by: (1) converting units, and (2) reclassifying soil types as World Resource Base Reference Soil Groups (IUSS Working Group WRB 2014), and (3) converting soil test values to a common method. For example, P-Olsen and P-Bray were never both measured in the same experiment. Therefore, published conversion factors was used to estimate P-Olsen from P-Bray 1: P-Olsen = 0.44 P-Bray 1 (Kleinman et al. 2001) and from P-Bray 2 to P-Olsen = 0.79 P-Bray 2 (Wuenscher et al. 2015). The database still included many missing values because soil descriptions and analytical procedures differed. A flowchart deducing different steps of estimating the missing data is presented in Supplementary Material 2 (Fig. S2). To handle the rest of the missing data on soil properties, we used the following approaches:

1. Pairwise correlation analysis was conducted to establish the correlation among paired soil properties. Paired soil properties with a Pearson correlation coefficient > 0.8 was selected. From this pair, the property with the highest number of missing values was dropped. Prior to dropping the property out, a linear equation was established and used to estimate the missing value of the retained property (with fewer missing values). But where the pair-wise points were both missing, the next approach was employed.
2. We used a Predictive Mean Matching (PMM) approach to impute the remaining missing soil data using the “*mice*” R-package (Van Buuren and Groothuis-Oudshoorn 2011). The PMM approach is based on regression analysis and estimates missing values by means of the nearest neighbor (Di Zio and Guarnera 2009; Vink et al. 2014). We used this approach so that the originality of the soil data and the underlying distribution are maintained (Little and Rubin 2002; Vink et al. 2014). Remaining missing values (18%) for soil pH, total C, Exch. K, silt and clay were estimated using the PMM approach.

Lastly, to calculate sampling variance for meta-analysis, we included the standard deviation (*sd*) in our database. If only the standard errors or coefficient of variation were reported, they were used to estimate the *sd*. In studies where no measures of variance were presented, a value of 1.5 times of the mean of all reported *sd*’s was assigned (Ishak et al. 2008; Ros et al. 2011).

### Database overview

The literature search on experiments conducted on smallholder farms yielded 503 publications on on-farm fertilizer maize experiments. We identified only 71 studies, which matched our criteria, with 202 observations for Kenya and 255 for other SSA countries (see Supplementary Materials 1, 2). The geographical distribution of the observations besides Kenya were; Togo 64, Uganda 49, Malawi 42, Tanzania 26, Zimbabwe 32, Nigeria 19, Ghana 12, Cameroon 8, Rwanda 2, and Benin 1. The experiments were limited from 1986 to 2016 and, include treatments with a single N application or a combined application of N with P and or K. Basal fertilizers were the commonly applied inorganic fertilizers (*e.g.,* di-ammonium phosphate and urea). Nitrogen application rates varied between 15 and 150 kg N ha^−1^ (Supplementary Material 1, Table S2). Wide ranges in maize yield between the fertilized and control treatments plots was observed (see Supplementary Material 1, Table S3). In general, the database contained higher control yields with more variability in Kenya compared to the rest of SSA countries.

Excluded studies include studies with little information on environmental and management factors (see Supplementary Material 2, Fig. S1). Information on soil properties was missing in 25% of the excluded studies. For example, only 45% of the studies reported on soil texture, CEC, Ca and Mg concentrations. Studies that reported aggregate data from multiple field trials (constitute 54% of excluded studies) were culled from the database and further analysis, since they did not record data on each trial sites. Studies which reported fertilizer treatments without including a control were also culled from our analysis and constituted 5% of the excluded studies. Most studies described one experiment on a single location. Only 31% of the observations in the dataset were extracted from studies with experiments at multiple locations, which had information on each of the individual site.

### Data analysis

First, dependent variables FR and N-AE were computed. The effect size (response ratio i.e. FR) estimator was used to quantify the magnitude of the effect of fertilizer application on yield (Hedges et al. 1999) and was considered a proxy index of soil responsiveness. Agronomic nitrogen use efficiency was taken to represent nutrient use efficiencies across the fertilizer studies. Secondly, a meta-analysis was conducted to: (1) quantify heterogeneity across fertilizer studies and, (2) to evaluate causes of variation in FR and effect size across categorical variables. Lastly, regression analysis was done to discern the continuous independent variables that explain variability in FR and N-AE.

#### Fertilizer response

Fertilizer response was taken as the ratio of the mean maize yield of the fertilized plot ($\bar{x}_{t}$ in kg ha^−1^) and the mean maize yield of the control plot ($\bar{x}_{c}$ in kg ha^−1^) (Hedges et al. 1999; Ros et al. 2011) and was computed as a natural log to normalize the data distribution (Johnson and Curtis 2001). A normalized FR is required to develop random effect meta-regression models. The *ln* FR was computed as:

$$ln \;{\text{FR}} = ln\left( {\frac{{\bar{x}_{t} }}{{\bar{x}_{c} }}} \right)$$

Soils with FR > 1 were categorized as responsive. Within the non-responsive soils (FR ≤ 1) we distinguished poor and fertile soils (less responsive), based on the maize yields in the control plots, and as described by Vanlauwe et al. (2014). The fertile soils category were soils where no significant increase in maize yield was realized after N fertilization or a combination of N with inorganic P or K, (Vanlauwe et al. 2014), but would still have high maize yields (> 1125 kg ha^−1^ for smallholder farm in SSA) as displayed in the control plots. A FR ≤ 1 meant that fertilization had no effect or negatively affected yield.

The sampling variance of the fertilizer response (FR^*v*^*)* is required to compute the heterogeneity between fertilizer studies and evaluate factors affecting FR. FR^*v*^ was calculated as:

$$ln \;{\text{FR}}^{V} = \left( {\frac{{\left( {sd_{t} } \right)^{2} }}{{n_{t} \left( {\bar{x}_{t} } \right)^{2} }} + \frac{{\left( {sd_{c} } \right)^{2} }}{{n_{c} \left( {\bar{x}_{c} } \right)^{2} }}} \right)$$

where *n* is the sample size/number of replicates, *sd*_*t*_ is the standard deviation for the yields within the treatment and *sd*_c_ is the standard deviation for the yields within the control.

#### Agronomic nitrogen use efficiency

The agronomic nitrogen use efficiency was computed following Vanlauwe et al. (2011):

$${\text{N-AE}} = \left( {\frac{{\bar{x}_{t} - \bar{x}_{c} }}{\text{FN}}} \right)$$

where ${\text{FN}}\; ( {\text{kg}}\;{\text{N}}\;{\text{ha}}^{ - 1} )$ is the amount of applied fertilizer N. The N-AE was reported as kg dry weight kg^−1^ N. The average N-AE was computed across the different groups of categorical factors (Table 1). We could not compute the sampling variance of N-AE, since N-AE is not an effect size as defined in the meta-analysis. Hence N-AE was not subjected to meta-analysis.

#### Meta-analysis of fertilizer response

We followed the methods used by Hedges et al. (1999) and Luo et al. (2006) to evaluate FR using a meta-analysis approach. The FR was used to evaluate soil responsiveness to N fertilization, or combinations with inorganic P and or K. To establish the different categories of soil responsiveness, we evaluated the relationship between FR and maize yield of the control plots. The dataset was split into three categories of soil responsiveness to fertilizer application, similar to Njoroge et al. (2017). To further evaluate these categories, we analyzed their corresponding soil properties.

To examine the heterogeneity (Q_T_) of FR in fertilizer studies across Kenya and SSA, a random effects (RE) meta-regression model was developed (Viechtbauer 2010). The RE model was fitted using the Restricted Maximum Likelihood method (Brown and Kempton 1994). A test of Q_T_ was used to assess how comparable the studies were and to test the significance of Q_T_ of the FR (Hedges and Olkin 1985). Significant Q_T_ of the FR indicates that the variation cannot only be attributed to the sampling error and other explanatory factors are playing a role as well (Huedo-Medina et al. 2006). The latter situation would provide an option to identify explanatory factors of the heterogeneity across fertilizer studies.

We tested the potential effect of publication bias in the meta-analysis using a regression test for the overall dataset (71 studies) (Viechtbauer 2010). The test is a quantitative representation of the importance of publication bias in the meta-analysis (Thornton and Lee 2000). The publication bias was also evaluated through a “*funnel*” plot. We analyzed the distribution of *ln* FR in the “*funnel*” plot to check if indeed publication bias was likely to influence the meta-analysis results (Viechtbauer 2010). The trim and fill method was used to estimate the number of additional observations necessary to change the results of the analysis from significant to non-significant (Duval and Tweedie 2000; Viechtbauer 2010).

To examine the influence of soil, climatic and management factors on FR, we conducted an analysis for the categorical variables, as a further step in meta-analysis (Table 1). The categorical variables included; soil types, soil textural classes, agro-ecological zones, type of management (farmer or researcher managed), range of N application rates and nutrient types (N, P and K). To compare the effect of fertilization across the groups, the weighted means $(ln \;{\text{FR}}_{w} )$ of FR and their corresponding 95% confidence intervals (CIs) were computed for each group, following Luo et al. (2006):

$$ln \;{\text{FR}}_{w} = \left( {\frac{{\mathop \sum \nolimits_{i = 1}^{m} ln \;{\text{FR}}\;w_{i} }}{{\mathop \sum \nolimits_{i = 1}^{n} w_{i} }}} \right)$$

where *i* is an observation, *w*_*i*_ is the weight of *i,* defined by the reciprocal of the *ln* FR^*v*^ (*w*_i_ = $1/ln \;{\text{FR}}^{v} )$, and *m* is the number of observations within a group of that categorical variable. The effect of fertilization for each group was considered significantly different from 1 if the CI did not overlap the line of no effect (*ln* FR = 0), and different from one another if their 95% CIs were non-overlapping (Hedges et al. 1999). A back-transformed $ln \;{\text{FR}}_{w}$ was reported in text and figures. The “*metafor*” R-package was used to conduct the meta-analysis (similar to Barto and Rillig 2010). Back transformed values (FR) were reported in the figures.

#### Regression analyses

To further study how soil properties, management and climatic factors (the continuous factors) affect FR and N-AE, general linear regression models (GLM) were developed. In this analyses *ln* FR or N-AE was the dependent variable and independent variables were: N application rate (only for *ln* FR), total C, soil pH, P-Olsen, exchangeable K, clay, silt and rainfall. The variables soil pH, total C, N application rates, rainfall, and P-Olsen were skewed to the left and were log-transformed to approximate normal distribution. Variables were standardized by dividing each observation with the standard deviation of the variable, so that each factor had equal representation in the GLM. The relationship between dependent (*ln* FR or N-AE) and independent variables was assessed based on the level of significance (*p*) and coefficient of determination (adjusted *R*^2^).

Further evaluation of the GLMs were conducted by computing the variable importance projections (VIP) scores from each GLM (*ln* FR or N-AE), which primarily indicate the relative measure of the importance of each predictors in the model (Kuhn 2008a, b). These scores were considered robust, because they took into account the orthogonal variation between independent factors (Chong and Jun 2005; Farrés et al. 2015) and high variation in *ln* FR or N-AE. The VIP scores were used to discern the important (key) factors, which also explain the underlying variation in FR or N-AE (Kuhn 2008a, b; Mehmood et al. 2012; Farrés et al. 2015). The scores are computed independently for each other (predictors) using the t-statistic (Kuhn 2008a, b). A criterion of VIP scores > 1 was adopted for identifying the important factors, so that those with scores > 1 were taken as the important ones (Chong and Jun 2005). The “pls” R-package was used for regression analysis (Mevik and Wehrens 2007). The “caret” R-package was used to compute VIP scores (Kuhn et al. 2014).

In the regression analysis of N-AE, we excluded N application rates as an independent variable. Inclusion of N application rate could have led to redundancy in the predictor information as it was used to compute N-AE.

## Results

### Fertilizer response

**Fig. 1:**
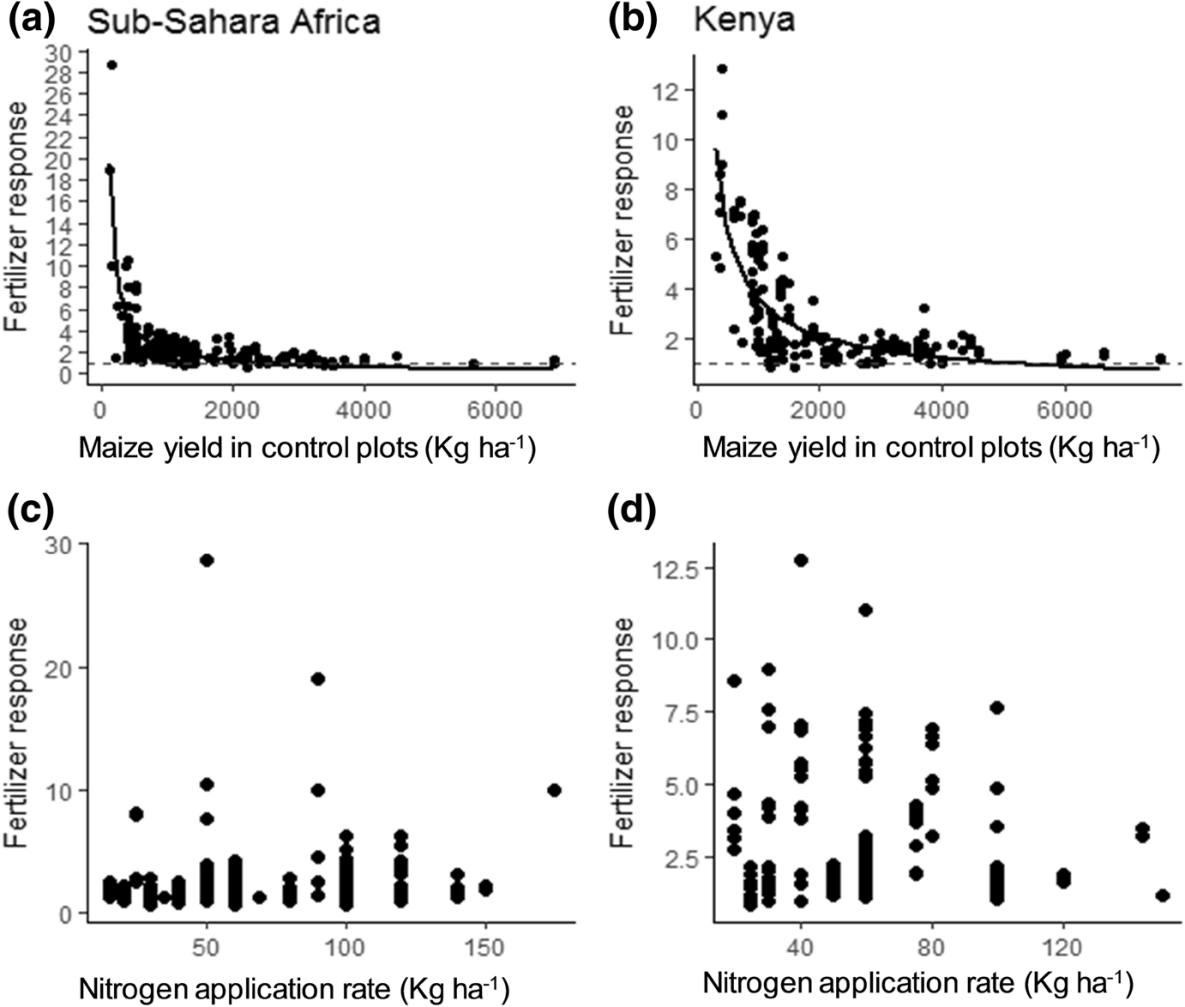
Fertilizer response (FR) as a function of maize yield in the unfertilized control plots (**a**, **b**) or N application rate (**c**, **d**) for Sub-Saharan Africa (**a**, **c**) and Kenya (**b**, **d**). The dashed line is the line of no response to the fertilizer (FR = 1). The solid lines describe non-linear relationships function as: FR = 32,244 (control yield)^−0.7^ (*P *= 0.003; R^2^ = 0.47) for Kenya and FR = 83 (control yield)^−0.5^ (*P *= 0.002; R^2^ = 0.49) for Sub-Saharan Africa

The maize yield almost doubled with N fertilization: The median FR was 1.8 for Kenya and 1.7 for SSA (excluding Kenya). There was a significant non-linear, negative relationship between FR and the maize yields of control plots (Fig. 1a, b) with *R*^2^ value of 0.47 for Kenya (*p* = 0.003) and 0.49 for SSA (*p *= 0.002). There was no obvious relationship between FR and N application rate (Fig. 1c, d) although the maximum attainable FR in Kenya tended to decrease with N application rate (Fig. 1d).

Responsive soils (FR > 1) were common in Kenya (86%) and SSA (89%). The non-responsive soils (FR ≤ 1) constituted 14% for Kenya and 11% for SSA. The maize yields in control plots of these non-responsive soils varied from 100 to 7000 kg ha^−1^. Of these soils, 72% were considered fertile non-responsive soils (control plots with maize yields > 1125 kg ha^−1^). At this point, the quadratic curve started to level, which was an indication of no significant effect of fertilization, and most observation (> 20%) were close to or below the line of no effect to fertilization (FR = 1, Fig. 1).

The mean FR was 2.2 for the responsive soils, 0.68 for poor, non-responsive and 0.89 for fertile, non-responsive soils in SSA including Kenya. The number of non-responsive soils for Kenya (51) was too small for further analyses, so we pooled the data of the non-responsive plots for Kenya and the rest of SSA for further analysis.

**Fig. 2:**
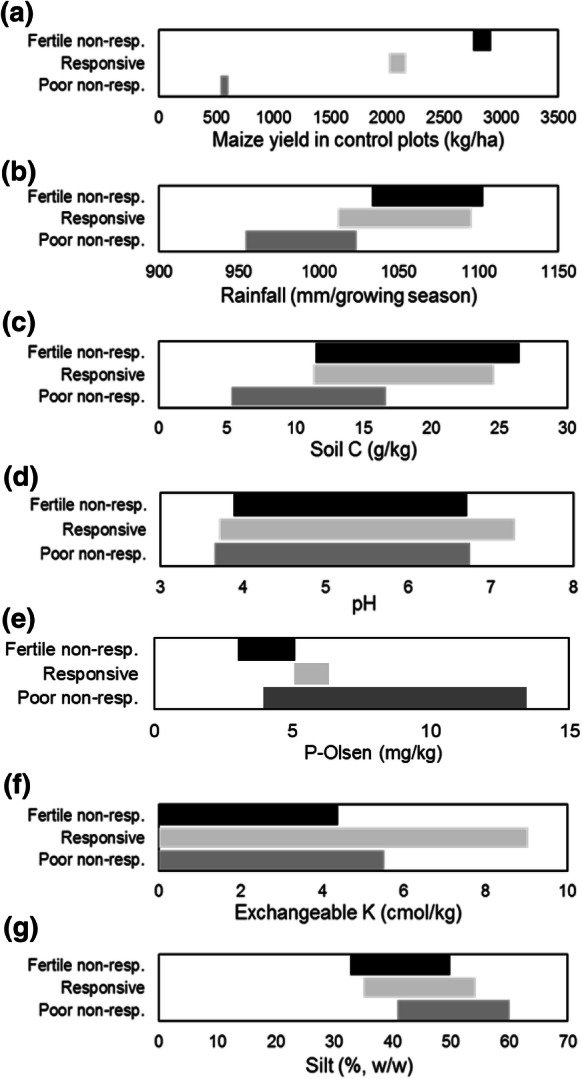
Ranges (mean ± 2 × SE) of maize in control plots, soil variables and rainfall for poor, non-responsive soils responsive soils and fertile, non-responsive soils

Soil characteristics varied within the three soil responsive categories (Fig. 2). For example, average total C ranged from 2 to 27 g kg^−1^ for poor, non-responsive soils and from 1 to 56 g kg^−1^ for fertile, non-responsive soils. The average total C content for responsive plots was 63% higher than that of poor, non-responsive plots. Surprisingly, the mean concentration of P-Olsen for the poor, non-responsive plots was higher (8.7 mg kg^−1^) than that of responsive soils (5.7 mg kg^−1^) and fertile, non-responsive plots (4.1 mg kg^−1^). Soil C and exchangeable K seemed to be the main separators between poor, non-responsive soils and the other two categories (Fig. 2c, f). The mean N application rates were on average 22% lower for poor, non-responsive plots compared to the responsive soils.

### Heterogeneity in fertilizer studies and test of publication bias

Random effect (RE) meta-regression model results indicate significant variation in FR among the observations of the fertilizer studies for Kenya (QT = 15,435, degree of freedom = 198, *p* < 0.001) and for SSA (QT = 1645, degrees of freedom = 245, *p* < 0.001). This implies independent variables explained a significant part of this variation other than the sampling error alone. Thus, evaluation of factors that attribute to the variability in FR was necessary.

The regression test results (z value = 0.75, *p *= 0.39) suggests absence of publication bias across the selected 71 fertilizer studies. Although the distribution of *ln* FR observations in the “*funnel*” plot was not symmetrical because of more relatively high values for *ln* FR, only 84 observation were missing and did not have any effect on the overall results of meta-analysis (Supplementary Material 1, Fig. S3). Additional observations would however have resulted in a more symmetrical “*funne*l” plot.

### Variability in fertilizer response

**Fig. 3:**
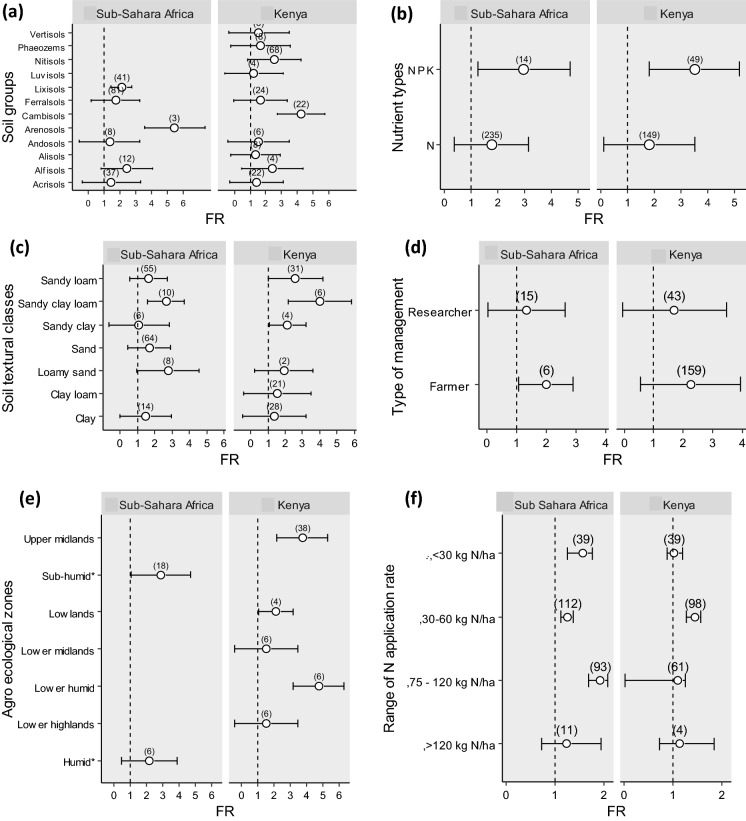
Means of the fertilizer response (FR) across categorical variables, **a** world reference soil groups, **b** nutrient types, **c** soil textural classes, **d** agro-ecological zones. The dashed line is the line of no response to the fertilizer (FR = 1). Error bars represent confidence intervals; numbers in brackets represent the number of observations

Weighted mean across categorical variables was used to assess variability between their sub-groups using CIs (Fig. 3). The meta-analysis showed that the CIs around the FR on of all soil orders except Cambisols (Kenya) and Areonsols (rest of SSA) overlapped with the line of no response. The FR was significantly higher than 1 for these two soil orders (Fig. 3a).

Combined application of N, P and K led to a doubling of the mean FR (*p *< 0.0001) both SSA and Kenya (Fig. 3b) compared to application of N alone. For plots in which only N was applied, the FR did not differ significantly from 1. Again, the variation in FR was large.

For Kenya, sandy soils in general tended to show a higher FR than non-sandy soils (Fig. 3c). This trend was, however, not confirmed for the rest of SSA. There, sandy loam soils were the only class of soils with a FR significantly higher than 1. For clay soils, the FR did not differ significantly from 1.

The FR was similar between the farmer and researcher-managed plots (Fig. 3d). The mean FR in SSA farmer-managed plots was significantly higher than 1.

The response to fertilization did not vary significantly among agro-ecological zones (Fig. 3e). In Kenya, the FR was highest in the lower humid zone (4.8) and > 1 also in the upper midlands and lowlands. There was no significant response to N fertilization in the lower midlands and lower highlands. For the rest of SSA, the mean FR for the sub-humid zone was 2.9. The FR_*w*_ for sub-humid zone was significantly higher than 1.

The FR had wide CI range (0.8–1.5) across the N application rates < 30 kg N ha^−1^ for Kenya (Fig. 3f). The average FR for 30–60 kg N ha^−1^ application ranges was 1.61 and was not significantly different from 1. For SSA, FR was not significantly different from 1 for N application rates range of 30–60 kg N ha^−1^ since the CI overlapped with the line of no effect.

### Agronomic nitrogen use efficiency

**Fig. 4:**
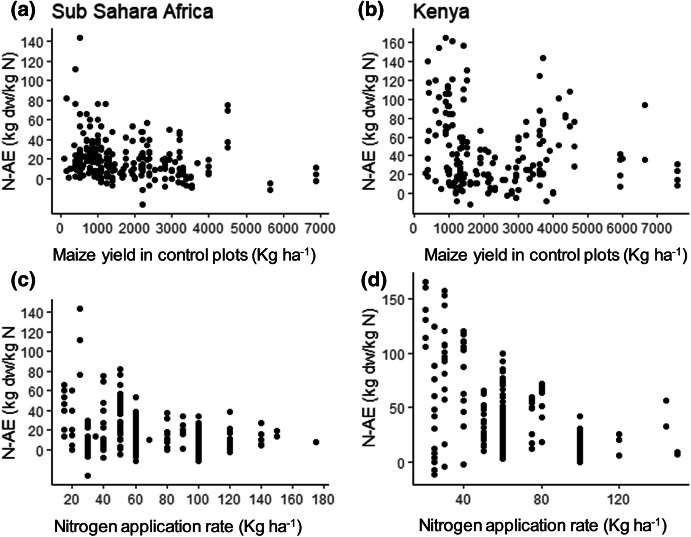
Agronomic nitrogen use efficiency (N-AE) as a function of maize yield of the control plots (**a**, **b**) or nitrogen application rate (**c**, **d**) across fertilizer studies for Sub-Saharan Africa (**a**, **c**) and Kenya (**b**, **d**)

The average N-AE was 42 kg dw kg^−1^ N for Kenya and 18 kg dw kg^−1^ N for the rest of SSA. The N-AE varied from − 27 to 165 kg dw kg^−1^ N across all observations (Fig. 4a, b). We did not find any significant relations between maize yield of the control plot (Fig. 4a, b) or N application rate (Fig. 4c, d) and N-AE, though the maximum attainable N-AE seemed to decline with increasing maize yields in the control plots and N application rates. Mean N-AE varied across the soil, climate and management factors (Table 2).

**Table 2:** Descriptive statistics of agronomic nitrogen use efficiency for categorical variables of soil and climatic factors

Factor	Level	Kenya			Sub-Saharan Africa		
		Mean	SE	n	Mean	SE	n
*Environmental*							
Soil order	Acrisols	17.3	4.9	22	20.0	2.5	37
	Alfisols	87.4	19.2	4	23.5	1.6	12
	Alisols	12.3	4.8	8	–	–	–
	Andosols	22.8	1.6	6	28.9	10.7	8
	Arenosols	–	–	–	15.3	2.2	3
	Cambisols	71.8	10.2	22			
	Ferralsols	42.0	8.0	24	16.7	2.1	81
	Lixisols	–	–	–	22.5	5.4	41
	Luvisols	10.5	3.0	4	–	–	–
	Nitisols	51.2	5.2	68	–	–	–
	Phaeozems	65.8	4.8	8	–	–	–
	Vertisols	22.1	4.6	6	–	–	–
Soil textural classes	Clay	26.7	6.1	28	19.5		14
	Clay loam	33.7	2.0	21			
	Loamy sand	20.8	–	2	22.5	4.7	8
	Sandy	–	–	–	10.8	0.7	64
	Sandy clay	20.4	2.2	4	3.5	9.6	6
	Sandy clay loam	54.6	21.6	6	17.2	5.9	10
	Sandy loam	49.8	7.0	31	16.8	3.1	55
Agro-ecological zone	Lower highlands 1	22.8	1.6	6	–	–	–
	Lower highlands 2	75.8	9.6	6	–	–	–
	Lower midlands	28.3	7.7	4	–	–	–
	Lowlands	20.4	3.5	4	–	–	–
	Upper midland	94.3	15.1	8			
	Humid	–	–	–	21.8	2.7	6
	Sub-humid	–	–	–	26.7	1.9	18
*Management*							
Manager	Farmer	44.6	3.2	159	18.3	1.4	234
	Researcher	32.9	4.7	81	22.4	1.6	63
Nutrient type	N, P, K	80.0	5.7	49	23.3		14
	N	30.4	2.5	149	18.1	1.4	235

–, missing statistic of the group; n, number of observations; SE, standard error

### Key factors affecting fertilizer response and agronomic nitrogen use efficiency

The regression (GLM) with the eight continuous predictors explained 29% of the variation in FR for Kenya and 13% for SSA (Table 2). Fertilizer response decreased significantly with increasing P-Olsen (*p *< 0.0001) in Kenya, but not in the rest of SSA. Here, soil pH, exchangeable K and average rainfall during a growing season were the significant predictors of variation in FR. They correlated positively with FR. Low values for soil pH and rainfall of a growing season displayed decreased FR (< 1). Fertilizer response increased marginally significantly with soil total C in Kenya (*p* = 0.10), but not in the rest of SSA. When we tested whether addition of maize yields of control plots as predictor could improve the predictive ability of the model, the adjusted R^2^ value increased from 29 to 63% for Kenya and 13 to 48% for SSA. In that case, P-Olsen no longer explained any variation in FR for Kenya, and for SSA rainfall and exchangeable K dropped out of the model. The FR decreased with increasing maize yield in the control plots (Fig. 1).

Eight continuous predictors were used to develop the regression model for N-AE. The best model explained 32% of variability in N-AE for Kenya and 3.5% for SSA (Table 2). Similar to FR, rainfall and total C positively influenced variability in N-AE and P-Olsen did so negatively in Kenya; for SSA variation in N-AE silt was the best predictor

**Fig. 5:**
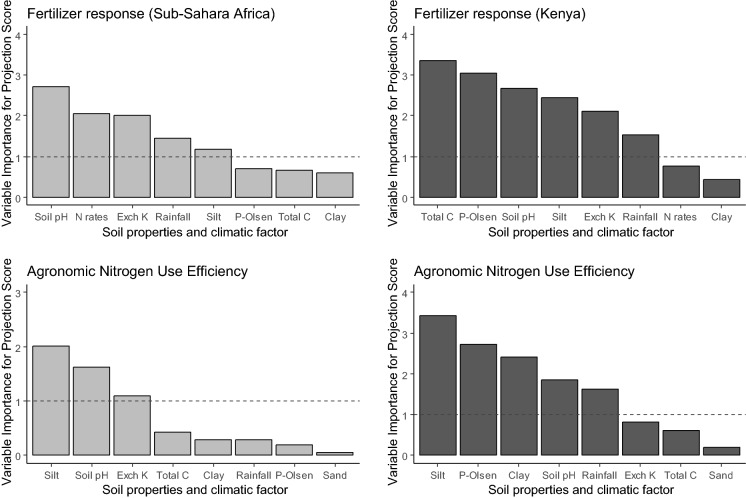
Relative importance of continuous management, soil and climate factors based on the variable importance projection (VIP) values computed from the general regression model explaining variation for in the fertilizer response (FR) (**a**, **b**) and agronomic nitrogen use efficiency (**c**, **d**). For Sub-Sahara Africa and Kenya. The dotted line represents the threshold value for the VIP value (VIP = 1) below which variables were considered not to be important predictors. *N rate* nitrogen application rate, *pH* soil pH, *P-Olsen* extractable phosphorus, *Total C* soil organic carbon and *Exch. K* exchangeable potassium

We identified key explanatory factors explaining variation in FR using VIP scores (Fig. 5). Three factors relevant to FR in both Kenya and the rest of SSA were exchangeable K, soil pH and rainfall (Fig. 5a, b). In addition, P-Olsen, total C and silt were relevant in Kenya, and N application rate in SSA. Clay was the least important factor for both Kenya and SSA. Results from the GLM indicate rainfall, as the significant factor influencing FR in Kenya and SSA. P-Olsen, total C and silt were additional significant factors for Kenya while soil pH and exchangeable K were significant for SSA (Table 3). These factors had VIP scores > 1 (Fig. 5). Nitrogen application rates was not significant but were important based on the VIP score that was 2.18 for SSA (Fig. 5).

**Table 3:** Regression model estimates of soil and climatic factors on fertilizer response and agronomic nitrogen use efficiency

*Fertilizer response*						
*R* ^2^	Adjusted *R*^2^	Predictor	Estimate	Standard error	*p* value	Significant level
Kenya (n = 202)						
0.29	0.26	Intercept	0.483	1.275	0.204	.
		Soil pH	0.166	2.679	0.008	**
		Log-Total C	− 0.024	− 3.353	0.001	***
		Log-P-Olsen	− 0.020	− 3.041	0.003	***
		Log-N rates	− 0.002	− 1.535	0.126	
		Log-Exch K	0.060	6.103	0.001	***
		Clay	0.001	0.425	0.671	
		Log-Silt	− 0.007	− 2.444	0.015	*
		Log-Rainfall	− 0.0008	− 0.771	0.004	*
Sub-Saharan Africa (n = 255)						
0.13	0.092	Intercept	− 0.221	− 0.734	0.464	
		Soil pH	0.154	2.714	0.007	**
		Log-Total C	− 0.006	− 1.649	0.101	
		Log-P-Olsen	0.021	3.049	0.003	*
		Log-N rates	0.002	2.043	0.042	*
		Log-Exch K	− 0.022	− 2.002	0.047	*
		Clay	− 0.002	− 0.591	0.555	
		Log-Silt	0.003	1.185	0.237	
		Log-Rainfall	− 0.0001	− 1.439	0.102	**

*Agronomic nitrogen use efficiency*						
*R* ^2^	Adjusted *R*^2^	Predictor	Estimate	Standard error	*p* value	Significant level
Kenya (n = 202)						
0.32	0.29	Intercept	52.652	22.943	0.023	*
		Soil pH	7.019	3.780	0.065	.
		Log-Total C	− 1.154	0.442	0.010	*
		Log-P-Olsen	− 1.098	0.404	0.007	**
		Log-Exch.K	3.429	0.589	0.001	**
		Clay	− 0.408	0.169	0.017	*
		Log-Silt	− 0.634	0.185	0.001	***
		Log-Rainfall	− 0.010	0.006	0.106	
Sub-Saharan Africa (n = 255)						
0.035	0.003	Intercept	11.183	11.791	0.344	
		Soil pH	1.697	2.283	0.458	
		Log-Total C	0.056	0.138	0.687	
		Log-P-Olsen	0.544	0.273	0.048	*
		Log-Exch.K	− 0.829	0.431	0.056	.
		Clay	− 0.013	0.113	0.912	
		Log-Silt	− 0.077	0.097	0.431	
		Log-Rainfall	− 0.001	0.004	0.781	

Significant codes: *** = 0.001; ** = 0.01; * = 0.05; . = 0.1

For N-AE, results of relative importance of key explanatory factors based on VIP indicate P-Olsen, clay, silt, soil pH, and rainfall as key determinates for Kenya (Fig. 5d). Only soil pH, exchangeable K and silt were the key factors explaining variation in N-AE for SSA: less silt meant higher N-AE (Fig. 5c).

## Discussion

### Factors affecting variability of FR and N-AE

Our results indicate that both FR and N-AE vary largely within Kenya, which supports the claim that fertilizer recommendations need to be refined to a higher spatial resolution. The FR response varied roughly from 1 to 12 (disregarding two extremes) and the N-AE from 0 to 160 kg dw kg^−1^ N. Fertilization, on average, almost doubled the maize yield in both Kenya and the rest of SSA. The average response was statistically significant only when N was applied in combination with P and/or K (Fig. 3b). The average N-AE we found (18 kg dw kg^−1^ N) for SSA is similar to that of Vanlauwe et al. (2011) who reported 19 kg dw kg^−1^ N for farmer-managed experiments. The average N-AE for Kenya (42 kg dw N kg^−1^) was substantially higher but not uncommon for East Africa (Vanlauwe et al. 2011).

Reoccurring variables significantly explaining variation in both FR and N-AE in Kenya and SSA are total C, pH, P-Olsen, exchangeable K, rainfall and silt (Figs. 2, 5, Tables 3). Soils with a lower pH (i.e. lower than the average of 5.2), less rainfall and less silt tended to have lower FRs and N-AEs. In line with earlier studies (Kihara et al. 2016), pH and FR and N-AE were positively related. This is probably because most soils in our study had a soil pH below the optimum of 5.5–6.5. At soil pH < 5.5 N mineralization rates decrease and P increasingly binds to the soil’s solid phase. Factors with a high VIP scores varied to some extent between Kenya and SSA, which must be related to differences in agro-ecological zones, soil orders and soil textural classes between these two regions. The results between factors that were significant from regression analysis and computed VIP scores also varied to some extent (Table 3, Fig. 5). For example, for SSA, silt was not significant based on the coefficient from the regression model (Table 3), but were important based on computed VIP scores (> 1, Fig. 5a). The variation in the results can be attributed to the difference in statistical computation. Grömping (2009) explained such computation differences, which is caused by the non-unique decomposition of model sum of squares in the regression model, due to correlated predictors. However, we used uncorrelated variables to develop the regression models, which is contrary to this observation. Unexpectedly, higher FR values (for N) were found in soils with higher P-Olsen concentrations (> 8.7 mg kg^−1^), but below the critical level of 15 mg kg^−1^. This may be attributed to the fact that in part of the entries, N fertilization was combined with P (and K) fertilization, and FR was > 1 particularly in those cases (Fig. 3b).

Nevertheless, it appeared difficult to capture a relevant amount of variation using parameters that are widely available. The meta-analysis of factors affecting FR provided few points of departure for spatial refinement of fertilizer recommendations. The wide CIs for soil orders and texture were all overlapping each other, although some orders and textural classes with FRs significantly > 1 were identified (the more fertile Cambisols and Arenosols for Kenya and SSA, respectively, and sandy (clay) loam soils consistently for Kenya and SSA). The regression analyses showed that the set of continuous environmental characteristics used, explained a very limited proportion of the variation in FR and N-AE (Table 3). The available continuous variables explained only 29% of the total variation in FR in Kenya and as little as 13% in SSA. For the N-AE the respective percentages of explained variation were even lower.

Units aggregating several factors determining FR or N-AE would intuitively be most suitable to refine fertilizer recommendations for spatially relevant units. The spatial mapping unit AEZ potentially captures a combination of factors (length of the growing season, climate, landform and soils) related to land use. As such, it aggregates some of the other individual variables tested and is currently used to refine fertilizer recommendations. However, the average FR in the AEZ distinguished for Kenya (not including soils but based on temperature, water supply and length of growing period; (Jätzold and Kutsch 2000) did not differ significantly (Fig. 4e), although the FR was significantly > 1 in three of the seven zones of Kenya. This renders AEZs an unsuitable unit for refining recommendations based on our results. Extending them with soil information (pH, P-Olsen, texture, order) could be a promising strategy.

The maize yield in the non-fertilized control seemed to be the best predictor for the FR (*p *= 0.0001; Fig. 1a, b). This variable can, similar to AEZ, be regarded an integral proxy for environmental (soil fertility, climate, weather), genetic (maize variety) and management factors (planting density, control of pests, weeds, diseases). Both for Kenya and for SSA the adjusted *R*^2^ increased substantially when these yields were added to the set of independent variables (from 29 to 63% for Kenya and from 13 to 48% for SSA). The FR was higher when the yield in the control plots was lower (Fig. 2a, b). However, this statistical relationship is probably a result of autocorrelation because the maize yield in the non-fertilized control (*x*_c_) is in the denominator of FR. This suggestion of autocorrelation is supported by the absence of any relationship between control yields and N-AE (Fig. 1a, b).

### Soil responsiveness to fertilizer application

The FR when considered as index, can provide useful tool for diagnosing soil responsive to fertilizer application. The majority of sites (> 85%) were responsive to fertilization for SSA and Kenya. To prevent complete failure of fertilizers, prior identification of non-responsive soils is of utmost importance. Non-responsiveness of poor soils is often related to low soil organic matter content (Tittonell and Giller 2013), causing soil physical constraints (low water-holding capacity), low micronutrient availability (Kihara et al. 2017) and low microbial activity leading to increased soil disease risk (Lal 2016). Our results confirm this: although the variation was high, the average C content of poor, non-responsive soils (11 g kg^−1^) was significantly lower (*p* = 0.031) than in responsive, (18 g kg^−1^) and fertile non-responsive soils (19 g kg^−1^; Fig. 2c). The soil responsiveness categories were clearly distinguished by total C and exchangeable K (Fig. 2c, f). Thus, total C and exchangeable K could act as useful indicators for discerning the different categories of soil responsiveness to N fertilization, which may be useful for nutrient management. The high variation indicates that non-responsiveness is a complex feature that is not easily operationalized using easily available environmental data. This is probably the reason that soil total C is not a powerful predictor of the FR (Table 3).

### Challenges for meta-analysis in agronomic studies

This study adhered to standards recommended for meta-analysis in agronomy studies (Philibert et al. 2012). However, exclusion of publications (only 71 studies out of the total of 503 found were acceptable) is a clear indication of the challenges for merging and comparing data from past literature for meta-analysis, which may be attributed to differences in reporting across fertilizer studies. For example, all studies reported on fertilizer treatments, which allowed us to quantify the FR and N-AE. Studies that did not report control treatments (5%; where no fertilization was done) were omitted from the analysis. Familiar environmental and management factors that influence FR were not reported. For example, there was variation on the different set of soil properties used in characterizing each study area. As a result, we imputed missing soil properties (18%), since different analytical methods were used for soil characterization.

There is therefore a need of promoting standards of reporting finding (results) in agronomy, specifically in fertilizer-related studies for future meta-analytical inferences. We recommend developing standard to provide enough information. For example, there should be a minimum list (set) of soil properties that should be included in future studies, and clear description of any other factors observed within the site that is under investigation. This may allow combination as well as comparability of datasets across agronomic studies. Supply of information describing the availed data (metadata) should be a requirement for all agronomic studies. However, developing guidelines, calls for detailed investigation that could avail a standard protocol of presenting additional information for agronomic studies similar to those developed for biochar and metabolic studies (Fiehn et al. 2007; Jeffery et al. 2011).

## Conclusions

The basic premise of this study was to identify key soil, climate and management factors that can be used to refine fertilizer recommendation across smallholder farms of SSA. The findings in this study indicate that available data layers can explain only very small amounts (< 33%) of variation in FR and N-AE and there is need for more systematic studies at high spatial resolution to identify yield-limiting factors. Our data indicated that soil pH, P-Olsen, exchangeable K, silt content and rainfall had significant but low levels of power in explaining variation in FR and N-AE. This finding implies that strategies for refining the current blanket fertilizer recommendation should include information on soil type, soil properties (texture, P-Olsen and total carbon). Such information can be derived through soil testing, which should be accompanied by nutrient response trials and preferably plant nutrient testing to diagnose limiting factors.

Due to the limitation of our dataset, this study did not comprehensively unravel factors that lead to soil non-responsiveness across smallholder farms. The complexity of soil responsiveness to fertilizer application requires further studies to fully understand other factors that led to non-responsive soils, besides total C, soil pH, exchangeable K and P-Olsen as indicated in this study.

## Electronic supplementary material

Below is the link to the electronic supplementary material.

Supplementary material 1 (DOCX 26 kb)

Supplementary material 2 (DOCX 125 kb)

## Abbreviations

AEZ: Agro-ecological zone
CI: Confidence interval
CEC: Cation exchange capacity
dw: Dry weight
FR: Fertilizer response
FR_w_: Weighted fertilizer response
FURP: Fertilizer use recommendation project
GLM: General linear model
IUSS: International union soil science
N-AE: Agronomic nitrogen use efficiency
PMM: Predictive mean matching
Q_T_: Heterogeneity
RE: Regression model
SSA: Sub-Sahara Africa
VIP: Variable importance in projection
