# Supplementary material for: Fertilizer response and nitrogen use efficiency in African smallholder maize farms
Source: Nutr Cycl Agroecosyst. 2018 Nov 15;113(1):1–19. doi: 10.1007/s10705-018-9958-y (PMC7357725; doi:10.1007/s10705-018-9958-y)
Supplement: Supplementary file 1 — Supplementary material 1 (DOCX 26 kb) [file 10705_2018_9958_MOESM1_ESM.docx]

| **Supplementary Material 1**  **Table S1** List of literature used to develop the database. | | | | |
| --- | --- | --- | --- | --- |
| **No.** | **Reference article** | **Journal name** | **Publication year** | **Volume, (Issue),Page No.** |
| 1 | Kayuki and Wortman | Agronomy Journal | 2001 | 93 , 929-935 |
| 2 | Ayoola and Adeniyan | African Journal of Biotechnology | 2006 | 5, 1336-1392 |
| 3 | Jama and Kiwia | Experimental Agriculture | 2009 | 45, 241-260 |
| 4 | Phiri et al. | African Journal of Agricultural Research | 2010 | 5, 1235-1242 |
| 5 | Baijuka et al. | Plant and Soil | 2006 | 279, 77-93 |
| 6 | Ikerra et al. | Nutrient Cycling in Agroecosystems | 2007 | 76 , 333-344 |
| 7 | Opala et al. | Nutrient Cycling in Agroecosystems | 2010 | 86, 317-329 |
| 8 | Mucheru-muna et al. | Agroforestry Systems | 2007 | 69 , 189-197 |
| 9 | Onyango et al. | Proceedings of the Seventh Eastern and Southern Africa Regional Maize Conference, 11–15 February. | 2001 | 330-334 |
| 10 | Mwangi | World Journal of Agricultural Sciences | 2010 | 3, 313-321 |
| 11 | Abunyewa et al. | Journal of Agronomy | 2007 | 6, (2), 302-309 |
| 12 | Olasantan et al. | Nutrient Cycling in Agroecosystems | 1997 | 46, 215-223 |
| 13 | Shisanya et al. | Soil & Tillage Research | 2009 | 103, 239-246 |
| 14 | Kaizzi et al. | Agricultural Systems | 2006 | 88, 44-60 |
| 15 | Jenssen et al. | Agricultural Water management | 2003 | 59,(3),217-237 |
| 16 | Gitari and Friesen | Proceedings of the Seventh Eastern and Southern Africa Regional Maize Conference, 11–15 February. | 2001 | 3, (9), 234-238 |
| 17 | Obaga et al. | KARI report | - | Unpublished |
| 19 | Fening et al. | African Journal of Environmental Science and Technology | 2009 | 59,(3),217-237 |
| 20 | Mucheru | Bationo et al (eds) Managing Nutrient Cycles to Sustain soil fertility in SSA | 2002 | 1,(4), 592003 |
| 21 | Mukuralinda et al. | Agroforestry Systems | 2009 | 80(211-221) |
| 22 | Mtambanengwe et al. | Nutrient Cycling in Agroecosystems | 2006 | 76,271-284 |
| 23 | Achieng et al. | Agriculture and Biology Journal of North America | 2010 | 3, 234-238 |
| 24 | Kimani et al. | Bationo et al (eds) Advances in Soil Fertility Management | 2007 | 15,111-126 |
| 25 | Smaling et al. | Agriculture, Ecosystems and Environment | 1992 | 80,211-221 |
| 26 | Macharia et al. | Journal of Animal & Plant Sciences | 2005 | 76,271-284 |
| 27 | Sakala et al. | Bationo et al (eds) Managing Nutrient Cycles to Sustain soil fertility in SSA | 2004 | 1(4), 430-439 |
| 28 | Okalebo et al. | Bationo et al (eds) Managing Nutrient Cycles to Sustain soil fertility in SSA | 2004 | 1(4), 360-372 |
| 29 | Nyongesa et al. | African Crop Science Conference Proceedings | 2009 | 41, 241-252 |
| 30 | Onyango et al. | KARI report | - | Unpublished |
| 31 | Macharia et al. | KARI report | - | Unpublished |
| 32 | Mathuva et al. | Field Crop Research | 1998 | 55, 57-72 |
| 33 | Jeranyama et al. | Agronomy Journal | 2000 | 92,239-244 |
| 34 | Kimetu et al. | Nutrient Cycling in Agroecosystems | 2004 | 68,127-135 |
| 35 | Amusan et al. | Nutrient Cycling in Agroecosystems | 2011 | 90, (3), 321-330 |
| 36 | Ssali | Fertilizer Research | 1990 | 23, (2), 63-72 |
| 37 | Akinnifesi et al. | Plant and Soil | 2007 | 294 (2), 203-217 |
| 38 | Kang et al. | Fertilizer Research | 1980 | 1, (2), 87-93 |
| 39 | Kaizzi et al. | Nutrient Cycling in Agroecosystems | 2006 | 88, 44-60 |
| 40 | Sangiga et al . | Plant and Soil | 1996 | 179,119-129 |
| 40 | Sangiga et al . | Biological Agriculture and Horticulture | 1986 | 3, 347–352 |
| 41 | Osiname et al. | Nutrient Cycling in Agroecosystems | 2000 | 56,209-217 |
| 42 | Gacheru and Rao | International Journal of Pest Management | 2001 | 47,(3), 233-239 |
| 43 | Casky et al. | Nutrient Cycling in Agroecosystems | 2002 | 243,1-10 |
| 44 | Nziguheba et al. | Plant and Soil | 2001 | 198,159-168 |
| 45 | Nyamangara and Nyagumbo | Nutrient Cycling in Agroecosystems | 2010 | 88, 103-109 |
| 46 | Adjei-Nsaih | Field Crop Research | 2007 | 103, 87-9) |
| 47 | Anyanzwa et al. | Nutrient Cycling in Agroecosystems | 2010 | 88, 39-47 |
| 48 | Fofana et al. | Nutrient Cycling in Agroecosystems | 2004 | 68, 213-222 |
| 50 | Esilaba et al. | Agricultural Systems | 2005 | 86,144-165 |
| 51 | Kihara et al. | Nutrient Cycling in Agroecosystems | 2011 | 90, 213–225 |
| 52 | Usiri et al. | Communication in Soil Science and Plant Analysis | 1998 | 27, (17&18), 2815-2828 |
| 53 | Mtambanengwe and Mapfumo | Plant and Soil | 2006 | 281, 173–191 |
| 54 | Sigunga et al. | Nutrient Cycling in Agroecosystems | 2002 | 62, (3), 263-275 |
| 55 | Mureithi et al. | Agroforestry Systems | 1994 | 27, (1), 31-51 |
| 56 | Nguu | Fertilizer Research | 1987 | 14, (2), 135-142 |
| 57 | Titonell et al. | Plant and Soil | 2008 | 313, (1), 19-37 |
| 58 | Msolla et al. | Nutrient Cycling in Agroecosystems | 2005 | 72, (3), 299-308 |
| 59 | Ayuke et al. | Bationo et al (eds) Managing Nutrient Cycles to Sustain soil fertility in SSA | 2004 | 1,(4), 65-77 |
| 60 | Nabuhungu et al. | Bationo et al (eds) Advances in soil fertility management | 2011 | 1, 325-335 |
| 61 | Nekesa et al. | Bationo et al (eds) Innovations as key to green revolution | 2011 | 2, 335-342 |
| 62 | Ssila et al. | Nutrient Cycling in Agroecosystems | 1990 | 23, (2), 63-72 |
| 63 | Vanlauwe et al. | Agronomy Journal | 2001 | 93,1191-1199 |
| 64 | Saidou | Agriculture, Ecosystems and Environment | 2003 | 100, (3),265-273 |
| 65 | Otinga et al. | Field Crop Research | 2013 | 140, 32-43 |
| 66 | Githinji et al . | Bationo et al (eds) Innovation as key to Green Revolution in Africa | 2011 | 2, 281-288 |
| 67 | Kathuku et al. | Bationo et al (eds) Innovation as key to Green Revolution in Africa | 2011 | 2, 265-270 |
| 68 | Kaizzi et al. | Agronomy Journal | 2012 | 104, 73–82 |
| 69 | Kamanga et al. | Experimental Agriculture | 2014 | 50, (2), 229-249 |
| 70 | Rusinamhodzi et al. | Field Crop Research | 2012 | 136, 12-22 |
| 71 | Kurwakumire et al. | Field Crop Research | 2014 | 164, (1), 136–147 |

**Table S2** Papers used in analysis, showing the country in which the experiment was conducted and nitrogen application rates

| **Reference** | **Country** | **N application rate (kg ha^-1^)** |
| --- | --- | --- |
| Kimani et al. (2007) | Kenya | 20,40, 60, 80, 100 |
| Kathuku et al. (2011) | Kenya | 20, 80 |
| Gitari and Friesen (2001), Smaling et al. (1992) | Kenya | 25, 50 |
| Szali (1990) | Kenya | 25,50, 100, 150 |
| Mwangi (2010) | Kenya | 30, 40, 50, 60 |
| Achieng et al (2010) | Kenya | 30 |
| Okalebo et al (2004), Onyango et al. (Unpublished) | Kenya | 30, 60 |
| Mathuva et al. (1998) | Kenya | 40 |
| Onyango et al. (2001), Shisanya et al. (2009), Obaga et al .(Unpublished), Mucheru (2002), Achieng et al. (2010), Macharia et al. (2005), Kimetu et al. (2004), Anyanzwa et al. (2010), Kihara et al. (2011), Githinji et al. (2011), Kathuku et al (2011) | Kenya | 60 |
| Macharia et al. (Unpublished) | Kenya | 60, 120 |
| Mureithi et al. (1994) | Kenya | 75, 100 |
| Nekesa et al. (2011) | Kenya | 75 |
| Sigunga et al. (2002), Titonell et al. (2000), Ngome et al. (2011) | Kenya | 100 |
| Ayuke et al. (2003) | Kenya | 120 |
| Achieng et al. (2010) | Kenya | 144 |
|  |  |  |
| Saidou (2003) | Benin | 60 |
| Nguu (1987) | Cameroon | 30, 60, 120 |
| Abunyewa et al. (2007), Adjei-Nsaih (2007) | Ghana | 60 |
| Fening et al. (2009) | Ghana | 90 |
| Sakala et al. (2004) | Malawi | 35, 69 |
| Casky et al. (2002) | Nigeria | 40 |
| Amusan et al. (2011) | Nigeria | 50, 100 |
| Ayoola & Adeniyan (2006), Olasantan et al. (1997) | Nigeria | 60 |
| Kang et al. (1980) | Nigeria | 80 |
| Vanlauwe et al. (2001) | Nigeria | 90 |
| Nabuhungu et al. (2007) | Rwanda | 50, 175 |
| Baijuka et al. (2006) | Tanzania | 50 |
| Usiri et al. (1991) | Tanzania | 60 |
| Jensen et al. (2003) | Tanzania | 140 |
| Fofana et al. (2004) | Togo | 20, 40, 50, 100 |
| Kaizzi et al. (2004) | Uganda | 40,80 |
| Kaizzi et al. (2012) | Uganda | 50, 80 |
| Kayuki & Wortman (2001), Esilaba et al .(2005), | Uganda | 80 |
| Jeranyama et al. (2000) | Zimbabwe | 60, 120 |
| Nezomba et al. (2010) | Zimbabwe | 90 |
| Nyamangara and Nyagumbo (2010) | Zimbabwe | 100 |
| Mtambanengwe et al. (2006), Mtambanengwe & Mapfumo (2006), Kurwakumire et al. (2014) | Zimbabwe | 120 |

**Table S3** Descriptive statistics of maize yields (Mg ha^-1^) used in the computation of the fertilizer response and agronomic nitrogen efficiency

| Location | n | Control treatments | | | |  | Fertilized treatments | | | |
| --- | --- | --- | --- | --- | --- | --- | --- | --- | --- | --- |
|  |  | Average | Standard deviation | Minimum | Maximum |  | Average | Standard deviation | Minimum | Maximum |
| Kenya | 202 | 2.3 | 1.6 | 0.3 | 7.6 |  | 4.4 | 2.1 | 1.0 | 11.8 |
| Sub-Saharan Africa | 255 | 1.8 | 1.2 | 0.1 | 6.9 |  | 2.9 | 1.5 | 0.3 | 8.3 |

n= number of observations (paired control and fertilizer application treatment)
