# Supplementary material for: Fertilizer response and nitrogen use efficiency in African smallholder maize farms
Source: Nutr Cycl Agroecosyst. 2018 Nov 15;113(1):1–19. doi: 10.1007/s10705-018-9958-y (PMC7357725; doi:10.1007/s10705-018-9958-y)
Supplement: Supplementary file 2 — Supplementary material 2 (DOCX 125 kb) [file 10705_2018_9958_MOESM2_ESM.docx]

1. Initial literature search

Supplementary Material 2: Additional analysis for the meta-analysis.

List of relevant reference & books

Studies = 39

Web of science

Studies = 203

Google Scholar

Studies = 154

Mendelely

Studies = 107

Total literature searched & found

Studies = 503

2. Screening of literature found

**Criteria**

- Is a maize monoculture?
- Is field located within African smallholder landscape?
- Is a randomized trial & mineral fertilizer applied as treatment?

Selection of potential relevant publication (studies) for the meta-analysis

**Excluded**

- No control reported,

Studies = 22

- No soil information,

Studies = 126

- Aggregated data from multiple trials, Studies = 234
- Intercrop of maize with other crops, Studies = 49
- Combined with inorganic fertilizer, Studies = 45

**Relevant Information**

- Control & fertilizer treatment
- Management factors
- Environmental factors

Final inclusion in the database

Studies = 71

(n = 457, total;

Kenya = 202, rest of SSA =255)

3. Database development

**Fig.S1**. Meta-analysis flow chart for selection of studies and database development, n represents the total number of observations. Control were considered as plots where no fertilizer (organic or inorganic) had been applied. Rest of SSA include other countries in SSA besides Kenya.

**Fig. S2**. Flowchart showing the different steps that were done to estimate the missing values for some of the soil properties in the database. The R^2^ is the Pearson correlation coefficient obtained from correlation analysis. Soil property that were highly correlated (R^2^>0.8), the property with the highest number of missing values was removed from the analysis.


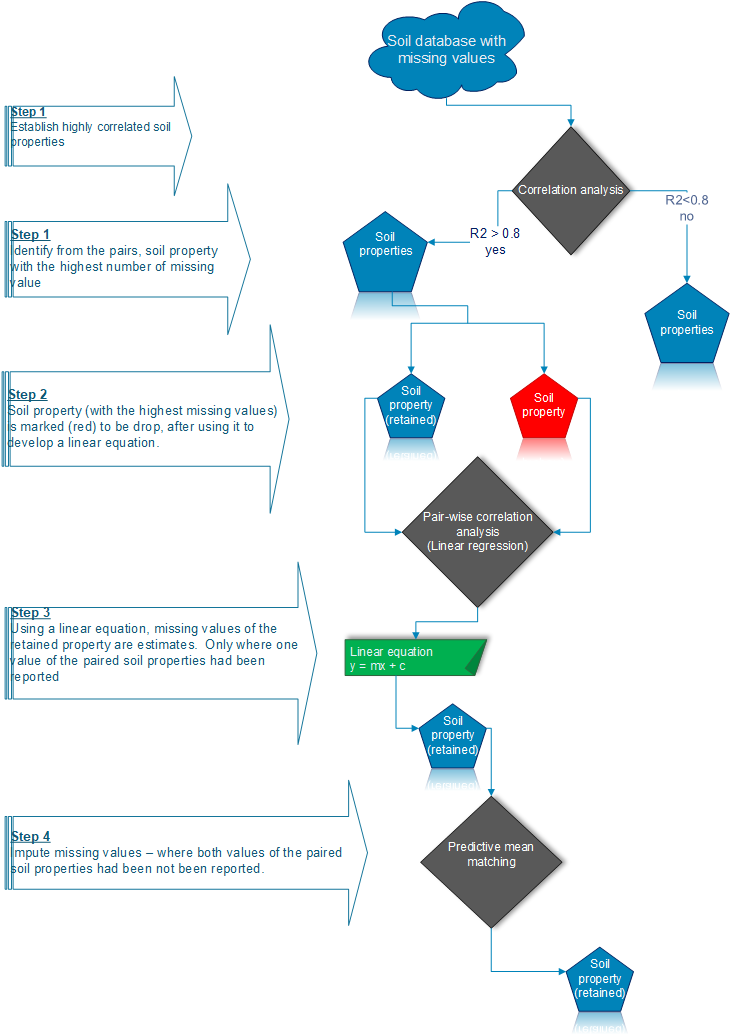


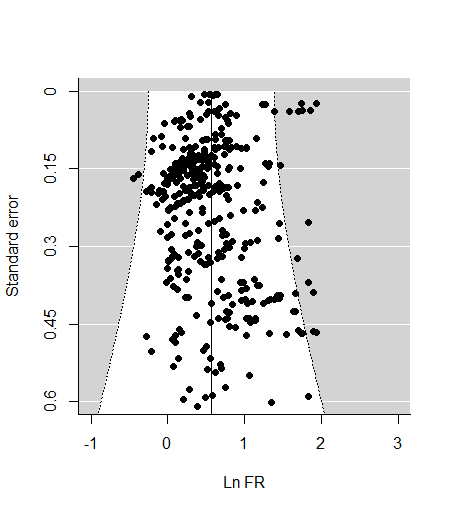


**Fig. S3**. Funnel plot showing the distribution of the natural log fertilizer response (*ln* FR) in the funnel plot adjusted with the heterogeneity of the random effect model. The vertical line is the regression line, which divides the funnel into equal portions to illustrate the asymmetry. Black dots are individual *ln* FR observations (n = 457); the grey area illustrates area outside the funnel
